# Analysis of the concept of caregiver role strain in informal caregivers: an approach according to Meleis

**DOI:** 10.15649/cuidarte.4000

**Published:** 2025-04-30

**Authors:** Bárbara Ebilizarda Coutinho Borges, Ana Clara Dantas, João Pedro Machado de Lima, Cyntia Leenara Bezerra da Silva, Maria Isabel da Conceição Dias Fernandes, Allyne Fortes Vitor

**Affiliations:** 1 Universidade Federal do Rio Grande do Norte. Natal, Brazil. barbara_ebilizarda@hotmail.com Universidade Federal do Rio Grande do Norte Universidade Federal do Rio Grande do Norte Natal Brazil barbara_ebilizarda@hotmail.com; 2 Universidade Federal do Rio Grande do Norte. Natal, Brazil. anaclaradaantas@yahoo.com.br Universidade Federal do Rio Grande do Norte Universidade Federal do Rio Grande do Norte Natal Brazil anaclaradaantas@yahoo.com.br; 3 Universidade Federal do Rio Grande do Norte. Natal, Brazil. pedro.lima.121@ufrn.edu.br Universidade Federal do Rio Grande do Norte Universidade Federal do Rio Grande do Norte Natal Brazil pedro.lima.121@ufrn.edu.br; 4 Universidade Federal do Rio Grande do Norte. Natal, Brazil. cyntialeenara@hotmail.com Universidade Federal do Rio Grande do Norte Universidade Federal do Rio Grande do Norte Natal Brazil cyntialeenara@hotmail.com; 5 Universidade Federal do Rio Grande do Norte. Natal, Brazil. isabel.dias@ufrn.br Universidade Federal do Rio Grande do Norte Universidade Federal do Rio Grande do Norte Natal Brazil isabel.dias@ufrn.br; 6 Universidade Federal do Rio Grande do Norte. Natal, Brazil. allynefortes@gmail.com Universidade Federal do Rio Grande do Norte Universidade Federal do Rio Grande do Norte Natal Brazil allynefortes@gmail.com

**Keywords:** Nursing Care, Caregivers, Caregiver Burden, Concept Formation, Atención de Enfermería, Cuidadores, Carga del Cuidador, Formación de Concepto, Cuidados de Enfermagem, Cuidadores, Fardo do Cuidador, Formação de Conceito

## Abstract

**Introduction:**

Caregivers provide assistance to people with physical, emotional, and cognitive problems; however, they often do not receive the necessary support, leading to the occurrence of Caregiver Role Strain.

**Objective:**

To analyze the concept of caregiver role strain theoretically and operationally to favor the instrumentalization of its use in research and clinical practice.

**Materials and Methods:**

This is an analysis of the concept of Caregiver Role Strain based on the Afaf Meleis model (2018). Operationalized in the following steps: (1) definition; (2) differentiation; (3) outlining of antecedents; (4) outlining of consequences; (5) modeling; (6) analogy, and (7) synthesis. A scoping review was performed to enable the execution of the four initial steps.

**Results:**

The literature review identified 67 studies capable of integrating the final sample. Based on its findings, a theoretical definition and empirical references of the concept were evidenced, in order to instrumentalize clinical inference. Furthermore, 29 antecedents and 21 consequences of the concept were recognized. Some attributes that differentiate Caregiver Role Strain from other similar concepts were also highlighted, such as a stressor that contributed to the suffering, the degree of extension of the patient's disabilities, the caregiver's perception of the patient's suffering, and the quality and quantity of the care burden to be provided.

**Discussion:**

Caregiver Role Strain can provide outcomes for informal caregivers and care recipients, manifesting worsening of symptoms, cognitive impairment, decreased quality of life, and physical, psychological, social, and financial aspects of both.

**Conclusion:**

A theoretical definition for Caregiver Role Strain emerges as a complex and multidimensional construct that includes subjective and objective factors, in which the demands of care, the impact of the patient's disease process, and the physical, psychological, social, and financial implications accompany a negative reaction to care-related activities. The analysis is useful in the process of inference and distinction of the concept of Tension and its similarities with synonymous terms, with the aim of adequately addressing the instruments used to observe the phenomenon, in an accurate and comprehensive manner, and the distinction from other commonly used definitions.

## Resumen

**Introducción:**

Los cuidadores brindan asistencia a personas con problemas físicos, emocionales y cognitivos, sin embargo, muchas veces no reciben el apoyo necesario, generando tensión en el rol de cuidador.

**Objetivo:**

Analizar teórica y operativamente el concepto de tensión del rol del cuidador para favorecer la instrumentalización de su uso en la investigación y la práctica clínica.

**Materiales y métodos:**

Se trata de un análisis del concepto Tensión del rol del cuidador basado en el modelo de Afaf Meleis (2018). Operacionalizado en los siguientes pasos: (1) definición; (2) diferenciación; (3) delimitación de antecedentes; (4) delimitación de consecuencias; (5) modelado; (6) analogía y (7) síntesis. Se llevó a cabo una revisión del alcance para permitir la ejecución de los cuatro pasos iniciales.

**Resultados:**

La revisión de la literatura identificó 67 estudios capaces de integrar la muestra final. A partir de sus hallazgos se evidenció una definición teórica y referentes empíricos del concepto, con el fin de instrumentalizar la inferencia clínica. Además, se reconocieron 29 antecedentes y 21 consecuencias del concepto. También se destacaron algunos atributos que diferencian el rol particular del cuidador en relación con otros conceptos similares, como el factor estresante que contribuyó al sufrimiento, el grado de extensión de las discapacidades del paciente, la percepción que experimenta el cuidador sobre el sufrimiento del el paciente y la calidad y cantidad de la carga asistencial que se le debe proporcionar.

**Discusión:**

La tensión del rol de cuidador puede proporcionar resultados para los cuidadores informales y los receptores de cuidados manifestándose empeoramiento de los síntomas, deterioro cognitivo, disminución de la calidad de vida y en los aspectos físicos, psicológico, social y financiero de ambos.

**Conclusiones:**

Surge una definición teórica de la Tensión del Rol del Cuidador como un constructo complejo y multidimensional que incluye factores subjetivos y objetivos, en el que las demandas de cuidado, el impacto del proceso de enfermedad del paciente y los recursos físicos, psicológicos, sociales y financieros acompañan un efecto negativo. Reacción a las actividades relacionadas con el cuidado. El análisis es útil en el proceso de inferir y distinguir el concepto de Voltaje y sus similitudes con términos sinónimos, con el objetivo de abordar adecuadamente los instrumentos utilizados para observar el fenómeno, de manera precisa e integral, y la distinción de otros comúnmente utilizados. definiciones.

## Resumo

**Introdução:**

Os cuidadores prestam assistência aquelas pessoas com problemas físicos, emocionais e cognitivos, todavia, muitas vezes não recebem suporte necessário convergindo para a ocorrência da Tensão do papel de cuidador.

**Objetivo:**

Analisar o conceito tensão do papel do cuidador teoricamente e operacionalmente para favorecer a instrumentalização do uso na pesquisa e na prática clínica.

**Materiais e métodos:**

Trata-se de uma análise do conceito Tensão do papel de cuidador fundamentada no modelo de Afaf Meleis (2018). Operacionalizada nas seguintes etapas: (1) definição; (2) diferenciação; (3) delineamento dos antecedentes; (4) delineamento dos consequentes; (5) modelagem; (6) analogia e (7) síntese. Uma scoping review foi realizada para viabilizar a execução das quatro etapas iniciais.

**Resultados:**

A revisão da literatura identificou 67 estudos capazes de integrar a amostra final. A partir de seus achados, evidenciou-se uma definição teórica e referenciais empíricos do conceito, a fim de instrumentalizar a inferência clínica. Além disso, houve reconhecimento de 29 antecedentes e 21 consequentes ao conceito. Ainda foram evidenciados alguns atributos que tornam a diferenciação da Tensão do papel de cuidador particular em relação aos demais conceitos similares, tais como um estressor que contribuiu para o sofrimento, o grau de extensão das incapacidades do paciente, a percepção experenciada pelo cuidador sobre o sofrimento do paciente e a qualidade e quantidade da carga de cuidado a ser prestado.

**Discussão:**

A Tensão do papel de cuidador pode proporcionar desfechos para os cuidadores informais e para os destinatários de cuidado manifestando agravamento dos sintomas, comprometimento cognitivo, diminuição da qualidade de vida e nos aspectos físicos, psicológicos, sociais e financeiros de ambos.

**Conclusões:**

Emerge uma definição teórica para a Tensão do papel de cuidador como um construto complexo e multidimensional que inclui fatores subjetivos e objetivos, em que as demandas de cuidado, o impacto do processo de doença do paciente e as implicações físicas, psicológicas, sociais e financeiras acompanham uma reação negativa às atividades relacionadas ao cuidado. A análise é útil no processo de inferência e distinção do conceito da Tensão e suas similaridades com termos sinônimos, com o intuito de abordar adequadamente os instrumentos utilizados para a observação do fenômeno, de forma acurada e abrangente, e a distinção das demais definições comumente utilizadas.

## Introduction

 The caregiver is sometimes treated as an informal professional, meaning that they represent a family member or friend who provides care to a sick, disabled or elderly individual in an unpaid manner. Such actions are integrated into daily life activities, in the management of medications for those in need, in addition to all the demands of care, as well as in the provision of financial and emotional support^1^.

 Caregivers help those with physical, emotional and cognitive problems, but often do not receive the necessary support. Therefore, the occurrence of burnout in the caregiver role can regularly result in psychological and physiological stressors, which negatively affect the personal health of the individual providing care. Many report manifestations of stress, physical fatigue, sleep disorders, financial worries and loss of social relationships, promoting social isolation and consequently the absence of a social support network that involves them^2^,^3^.

 In this context, the term burden is commonly used, which is defined as any reduction in quality of life attributed to caregiver burden or stress involved in caregiving. More specifically, caregiver burden corresponds to a negative psychological and physical state that emerges during the caregiving process for patients or those who, for some reason, are unable to care for themselves. For this reason, based on this state, it is possible to understand when the care provided can have negative effects on the physical, financial and mental health of the individual^3^,^4^.

 The terms overload and stress related to the caregiver role are commonly considered synonymous, as they present similar definitions and relate to the emotional and physical transformations in the caregiver's experience, and are therefore understood as a multidimensional concept of care provision^5^. Although they are understood in a similar way, it is necessary to distinguish the threshold of the theoretical content presented by each of them, since the high burden of the caregiver will include quantitative levels of stress on their health. It is then clear that the Stress of the caregiver role may not be well clarified, as well as its theoretical definitions and relationships of similarities or the best way to infer it.

 The study of concept analysis can reinforce professional communication regarding the phenomena that surround them. Therefore, it enables the analysis of the substantial elements of the concepts, while also clarifying obscure idealizations and promoting the establishment of hypotheses that represent the relationships involved in the chosen concept^6^. Phenomena are perceived and only when organized and labeled do they become concepts^6^,^7^.

 Concept analysis can be performed when a concept has already been introduced and defined in the literature of its specific discipline. From this, it demands the need to propose an additional study to favor the advancement to the next level of development, thus making it more applicable by translating the theoretical content into disciplinary research and clinical practice, expanding the understanding among professionals who know it or even favoring its verification with greater clarity^7^,^8^.

 In a previous study, it was possible to recognize Caregiver Role Strain as an unwanted human response of the caregiver^9^. However, it was not possible to deconstruct it in its essence and reconstruct it to promote nursing lexical knowledge or to contribute to instrumentalizing theoretical development and testing, since this was not the objective of the study. For this reason, the phenomenon is part of a pertinent concept in the scope of clinical practice and health research.

 When considering the complexity of the burden of care and the existence of various disciplinary concepts like Caregiver Role Strain, theoretical confusion arises, and there is a need to clarify this conceptual gap. In the absence of a clear differentiation between the construct of strain and similar terms, communication between researchers and care professionals will not be well defined and the synthesis of scientific knowledge may be challenging. In addition, the imprecision of its conceptual and operational definitions remains as unresolved gaps^10^.

 For all these reasons, it is pertinent and necessary to clarify this concept as a step towards the improvement and construction of theoretical and operational knowledge, since conceptual clarity is seen as a means of translating empirical science to the practical world and through concept analysis the researcher is able to identify the group of attributes that constitute the concept^10^.

 Therefore, the choice of the model proposed by Meleis^7^ occurs due to the need to list conceptual and operational definitions that characterize Caregiver Role Strain and to propose an additional study to favor advancement to the next level of development of the concept, thus making it more applicable by translating the theoretical content into disciplinary research and clinical practice. And through the steps recommended by the author, it is feasible to differentiate similar concepts, identify empirical references, antecedents and consequences and thus visualize the conceptual application from an analogy and case structures to list their respective conceptual and operational definitions around the phenomenon.

 Therefore, the research is justified by the demand to offer a concept analysis that addresses the Stress of the caregiver role, with the aim of contributing to theoretical development, based on knowledge of theoretical and operational definitions, empirical references capable of measuring the phenomenon and similarity relations, enabling the translation of this knowledge. Therefore, the study aims to analyze the concept theoretically and operationally to favor the instrumentalization of its use in research and clinical practice.

## Materials and Methods

This is an analysis of the concept of Caregiver Role Tension based on the Afaf Meleis model (2018)^7^. To this end, it was operationalized in the following stages: (1) definition; (2) differentiation; (3) outlining of antecedents; (4) outlining of consequences; (5) modeling; (6) analogy; and (7) synthesis. 

To enable the execution of the four initial proposed stages, a scoping review was carried out, structured according to the recommendations of the JBI Manual for Evidence Synthesis^11^. Its search protocol was constructed according to the Preferred checklist Reporting Items for Systematic Review and Meta- Analysis Protocols and registered in the Open Science Framework (OSF) platform, with the corresponding Uniform Sequential Identifier Resource Locator (https://osf.io/gyva3/). The research dataset was stored in Mendeley Data, a specific repository for this purpose^12^. 

For this review, the following steps were developed: formulation of the research question; identification of relevant studies in the scientific literature; selection of studies; data mapping; and synthesis of results. 

In the first stage, the following questions were raised: what are the theoretical and operational definitions capable of defining the concept? What are the empirical references capable of observing and measuring the concept? What are the similarities and differences between the concept and similar concepts? What are the consequences of the concept in literature? 

The second stage of the review corresponded to the selection of studies in the databases and for this purpose five of them were listed, namely: SciVerse Scopus (Elsevier), MEDLINE/ PubMed (via National Library of Medicine), WEB OF SCIENCE, SAGE Journals Online and PsycINFO (APA). Then the studies present in Google® Scholar were retrieved, composing the second stage of the search, in this phase the Publish software or Perish (https://harzing.com/resources/publish-or-perish) was necessary to retrieve and analyze the recognized findings. Subsequently, in the third stage, there was a manual search in the list of references for the research included in the other phases of the study.

To support the search in the databases, the following descriptors indexed in Medical Subject were used Headings (MeSH): “Caregivers”; “Caregiver”; “Caregiver Burden”; Concept Formation and Conceptualization, with the addition of the keywords: “Family caregiver”; “Informal caregiver ”; “Caring relationship ”; “ Care dependency ”; “Stress”; “Overload” and “Voltage”. The search crossing was performed using the Boolean operators OR and AND. 

The inclusion criteria defined were: complete studies available in the data sources; presentation of the concept of Caregiver Role Strain; and studies that identified theoretical and operational definitions, similarities, consequences or antecedents of the concept. Editorials, letters to the editor and abstracts were excluded. No time frame or language limit was proposed, with the purpose of recognizing a greater breadth of studies, given that the concept analysis aims to broadly review the literature on the phenomenon. 

In the identification phase, the third stage, those studies recognized through searches were captured and exported to the Rayyan – Intelligent software Systematic Review (https://rayyan.ai/) in December 2023 to identify duplicates, select, include or exclude studies. Two researchers, under active blinding, read the titles and abstracts of the studies and then proceeded to assess the effectiveness of the eligibility criteria to determine the initial screening of the sample. If necessary, the third researcher would be designated to resolve apparent discrepancies. It is mentioned that duplicates were counted only once. After refining the studies by the inclusion criteria, the included articles were read in full in a rigorous manner to assertively identify the valid studies and proceed to the data collection stage. 

Data mapping is the fourth stage of the review, and for this reason, a data extraction instrument structured in Microsoft Excel 2019®, built for this purpose, was used. For the included studies, the variables related to the research identification data were identified and extracted, namely: authors, title, year of publication, data source, country, language and objective, in addition to methodological data and the elements to compose the concept analysis, such as: operational and conceptual definitions, empirical references, antecedents, consequences and similarities, which were assessed according to the stages proposed by Meleis . 

The studies were also assessed according to the levels of evidence presented, according to the classification of Polit and Beck^13^. They are: level I - systematic review/meta-analysis of Randomized Controlled Trials (RCTs); level II - RCTs; level III - non-randomized (quasi-experimental) trials; level IV - systematic review of non-experimental studies; level V - non-experimental/observational study; level VI - systematic review/meta-analysis of qualitative studies; level VII - qualitative/descriptive study; level VIII - source unrelated to the research (internal evidence and expert opinion). 

Finally, in the fifth stage of the review, the synthesis of the results was presented descriptively and, according to the findings of the sample, established in the form of tables and figures. And for the construction of the modeling, analogy and synthesis stages, the pertinent findings of the literature review were used. In the construction of a similar model, as it was intended to illustrate the concept in its entirety, the predecessor characteristics and the consequences of Caregiver Role Strain were prevalent. 

As this was a study using secondary data available in the literature, there was no need for ethical assessment. 

## Results

**Figure 1:**
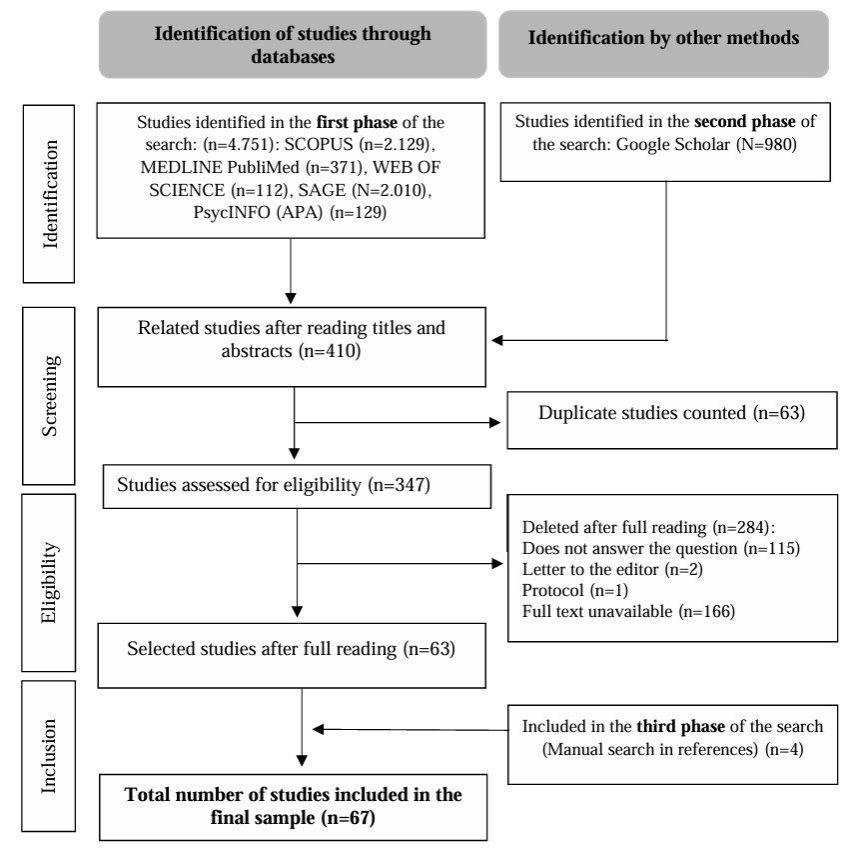
Flowchart of search and selection of studies. Natal, 2024

A total of 4,751 studies were identified in the first phase of the scoping review. In the second phase, 980 were identified in Google Scholar. After reading the titles and abstracts and accounting for duplicates, 347 studies were assessed for eligibility. After applying the inclusion and exclusion criteria, 63 were selected. Subsequently, four studies were included in the manual search of the reference lists, totaling a final sample of 67 studies. The flowchart of the search and selection of studies in the data sources is shown in Figure 1. 

The scoping review included 67 studies in its sample. According to the presentation in Table 1, the characterization data of the included studies can be observed, according to the data source, continent, language, year, study design and level of evidence. 

**Table 1:** Sociodemographic characteristics, personal and work history (n=288)

Variables	n (%)
Data source	
Google Scholar	44 (65.67)
MEDLINE/ PubMed	8 (11.94)
SAGE Journals	6 (8.96)
SciVerse Scopus	4 (5.97)
Reverse search	4 (5.97)
PsycINFO	1 (1.49)
Continent	
Europe	22 (32.84)
America	21 (31.34)
Asia	21 (31.34)
Africa	2 (2.99)
Oceania	1 (1.49)
Language	
English	66 (98.51)
Portuguese	1 (1.49)
Year	
2019-2023	39 (58.21)
2012-2018	14 (20.90)
2002-2008	7 (10.45)
1990-2001	7 (10.45)
Study design	
Transversal	34 (50.75)
Systematic Review	12 (17.91)
Methodological	12 (17.91)
Longitudinal	5 (7.46)
Mixed	3 (4.48)
Clinical Trial	1 (1.49)
Level of evidence	
V	52 (77.61)
I	12 (17.91)
IV	1 (1.49)
III	1 (1.49)
II	1 (1.49)
Total	67(100)

According to the findings shown, the most prevalent data source was Google Scholar (65.67%). In relation to the continent, Europe has a slight predominance of research (32.84%), followed by America and Asia, making up 31.34% of the percentage of studies.

The most common language was English (98.51%) and the years 2019-2023 (58.21%) highlight the rise in research on the concept in the last five years. In addition, it is observed that 50.75% of the studies assume a cross-sectional design, therefore, it can be reflected in the need to encompass distinct and varied approaches. Finally, the predominant level of evidence is V (77.61%), followed by level I (17.91%).

**Definition**

The process of defining contributes to theoretical clarification, to outline sub concepts and dimension the concept, therefore, the definition stage employed depends on the label given to the phenomenon. To this end, it is necessary during this stage to identify theoretical and operational concepts, so that ambiguities can be clarified and their relationships with empirical references can be made viable^7^.

In this sense, a diversity of theoretical and operational definitions for Caregiver Strain were evidenced in the research, in which 80,5% of the studies exhibited theoretical definitions capable of encompassing historical perspectives and conceptual evolution, with attributes that define it in its multidimensionality and as a multifactorial phenomenon. Thus, they encompass the physical and psychological aspects of the individual who cares or understands it as an adverse effect that has repercussions on emotional, social, financial, physical and spiritual functioning.

In addition, the findings also demonstrate recurring mention and distinction between objective and subjective burdens, with the objective burden referring to the observable consequences on the physical and psychological well-being of the caregiver due to caregiving assistance, and the subjective burden reflecting the psychological reactions to the care experience.

In summary, based on the results shown, the following theoretical definition for Caregiver Role Strain emerges: “Caregiver Role Strain is a complex and multidimensional construct that includes subjective and objective factors, in which the demands of care, the impact of the patient's disease process and the physical, psychological, social and financial implications accompany a negative reaction to care-related activities”.

Regarding operational definitions, the application of instruments, interviews with open questions and self-reports by caregivers were provided. Knowing that such definitions must be referenced and contextualized in care practice, and that from this it is feasible to use them in health care, the study enabled the identification of the main empirical references used in the recognition of caregiver stress. Thus, there are 39 predominant instruments, as shown in Table 2.

**Table 2:** Empirical references capable of measuring caregiver role strain according to the Scoping Review. Natal, 2024

Empirical References*	n(%)
Zarit Burden Interview (ZBI)	19 (28.36)
Open-ended question instruments constructed	8 (11.94)
Caregiver Burden Inventory (CBI)	6 (8.96)
Caregiver Burden Scale (CBS)	6 (8.96)
Caregiver Strain Index (CSI)	6 (8.96)
Caregiver reaction assessment (CRA)	3 (4.48)
Montgomery Borgatta Caregiver Burden Scale (MBCBS)	3 (4.48)
Family burden interview schedule (FBIS-24)	2 (2.99)
Burden Interview (BI)	2 (2.99)
Screen for Caregiver Burden (SCB)	2 (2.99)
Burden Scale for Family Caregivers (BSFC)	2 (2.99)
Burden Assessment Scale (BAS)	1 (1.49)
Family Burden Scale (FBS)	1 (1.49)
Self-Perceived Pressure by Informal Care Scale	1 (1.49)
Berlin Inventory of Caregiver Stress-Dementia (BICS-D)	1 (1.49)
Modified Caregivers Strain Index (CM-CSI)	1 (1.49)
Family Strain Questionnaire-Short Form (FSQ-SF)	1 (1.49)
Multidimensional Caregiver Strain Index (MCSI)	1 (1.49)
Pearlin's Caregiving Stress Questions tionnaire	1 (1.49)
Perceived Caregiver Burden Scale (PCB)	1 (1.49)
Picot Caregiver Rewards Scale (PCRS)	1 (1.49)
Bakas Caregiving Outcome Scale (BCOS)	1 (1.49)
Parental Stress Scale (PSS)	1 (1.49)
Caregiver reaction assessment (CRA)	1 (1.49)
Montgomery Borgatta Caregiver Burden Scale (MBCBS)	1 (1.49)
Spanish version of Caregiver Burden Inventory (CBI)	1 (1.49)
Brazilian version of the Burden Interview (BI)	1 (1.49)
Burden Scale for Family Caregivers (BSFC)	1 (1.49)
Bakas Caregiving Outcomes Scale (BCOS)	1 (1.49)
Short version of Burden Scale for Family Caregivers (BSFC-s)	1 (1.49)
Caregiver Burden General Strain Index (CB-GSI)	1 (1.49)
Caregiver Distress Scale (CDS)	1 (1.49)
Relative Stress Scale	1 (1.49)
Family Caregiving Inventory;	1 (1.49)
Caregiver Distress Scale (NPI-CD)	1 (1.49)
The Multidimensional Caregiver Strain Index (MCSI)	1 (1.49)
Caregiver Strain Questionnaire (CGSQ)	1 (1.49)
The Caregiver Burden Scale in the End-of-Life Care (CBS-EOLC)	1 (1.49)
Palliative Performance Scale – PPS	1 (1.49)

*Multiple answers

The findings show that the most used instruments, therefore, those that were most prevalent in the observation and measurement of the phenomenon of caregiver strain were the Zarit Burden Interview (ZBI) (28.36%), Open-ended question instruments constructed for this purpose (11.94%), Caregiver Burden Inventory (CBI) (8.96%), Caregiver Burden Scale (CBS) (8.96%) and Caregiver Strain Index (CSI) (8.96%). These empirical references were applied in different care contexts, involving acute diseases, disabilities and chronic health conditions.

**Differentiation**

Regarding the differentiation stage, it involves the process of classifying and separating similarities and differences between the concept and its analogues. In this sense, the review identified different concepts that can be used as terms equivalent to tension, therefore, they have similar definitions and applicability. However, they are not considered synonyms, and it is necessary to distinguish their meanings, as well as to establish the conceptual limits of each one^7^. 

The search resulted in the following terms like caregiver strain, namely: overload (28.36%), burden (17.91%), stress (16.42%), caregiver load (11.94%), caregiver burden (11.94%), strain (10.45%), role strain (7.46%), exhaustion (2.99%), caregiver burnout (1.49%), emotional exhaustion (1,49%) and primary caregiver syndrome (1.49%). 

However, it is understood that the meanings assumed by each one present points of convergence and divergence, demanding the need for individual clarification, so that they can be applied appropriately in each situation experienced by the caregiver. Linked to this, a greater degree of familiarity is perceived between the definitions of overload, burden or caregiver burden and load. The comparison between the definitions evidenced in the study is arranged as shown in Table 3. 

**Table 3:** Theoretical definition of similar concepts of Caregiver Role Strain. Natal, 2024

Similar concepts	Theoretical definition
Overload	"The perception of the degree to which physical health and psychological well-being, social life and financial situation are affected by illness."
Caregiver's burden	"The impact of the psychological, physical, financial and social demands of caregiving may not be culturally appropriate because it may carry negative connotations about the role of the family caregiver."
Caregiver burden	"A dynamic and multidimensional reaction that results from an imbalance of care demands and resources, and induces overload in one or more of the four components: physical, psychological, social and financial issues."
Stress	"A subjective reaction to an assessment of demands."
Caregiver burnout	"Progression of the burden, in which the experience of care is no longer a healthy option for the caregiver or the recipient of care, given that when there is an increase in the burden of care it has repercussions on their actions and consequently will affect the recipients of care."

Source: research data.

Thus, burden is defined as “ the perception of the degree to which physical health and psychological well-being, social life and financial situation are affected by illness”^14^. Caregiver burden involves the impact of the psychological, physical, financial and social demands of care; at the same time, the burden may still not be “culturally appropriate, because it may carry negative connotations about the role of the family caregiver.”^15^. “Caregiver burden” refers to the impact of caregiving on caregivers, “a dynamic and multidimensional reaction that results from an imbalance of care demands and resources, and induces overload in one or more of the four components: physical, psychological, social and financial issues.”^16^. 

Stress is perceived as “ a subjective reaction to an assessment of environmental demands”^17^. Caregiver burnout is understood as a progression of the burden, in which the experience of care is no longer a healthy option for the caregiver or the care recipient, given that when there is an increase in the care burden, it has repercussions on their actions and consequently affects the care recipients and other members of the lived context^18^. 

The concepts of stress were initially defined and applied in the field of mechanical engineering a long time ago. However, stress and strain are distinct, although they are related. And when checking the measures of stress, strain and load used in health research, it is found that the terms are used interchangeably, without a commonly accepted definition^19^. 

In general, all concepts highlighted by the research corroborate the understanding of the use of the terms as synonyms and justify the ambiguity in the use of each one, given the similarities in the manifestation of the phenomenon. However, the concept of overload appears to be the main confounder, with tension sometimes being identified as a symptom of this phenomenon. 

Therefore, some attributes are highlighted that differentiate the Stress of the caregiver role from the other concepts mentioned above, such as a stressor that contributed to the suffering, a dynamic and multifaceted reaction depending on the degree of coping or resilience in the face of the demands of care, the degree of extension of the patient's disabilities, the relationship between the roles, the perception experienced by the caregiver about the patient's suffering and the quality and quantity of the care burden to be provided. It is known from this that Stress can be observed as a manifestation of physical signs and symptoms caused by the burden of care or by the responsibility of maintaining the well-being of the family member. 

**Outline of antecedents**

In the antecedents outlining stage, one must understand the context that precedes the occurrence of the concept, the conditions and/or factors capable of predicting the onset of Caregiver Role Strain. In this sense, the purpose is to try to define the contextual conditions under which the concept is perceived and is expected to occur^7^. To this end, this review recognized the factors as shown in Table 4. 

**Table 4:** Antecedents of Caregiver Role Strain according to the Scoping Review. Natal, 2024. (n= 67)

Background*	n(%)
Care hours spent^17^,^19^,^20^–^37^	20 (29.85)
Disease severity^10^,^21^,^22^,^26^,^27^,^29^,^38^–^49^	19 (28.36)
Increased functional or cognitive impairment of the patient^16^,^18^,^26^-^29^,^31^,^38^-^40^,^47^,^48^,^50^-^52^	16 (23.88)
Type of relationship with care recipient^17^,^25^,^34^,^39^,^43^,^53^-^59^	12 (17.91)
Dependency^16^,^18^,^24^,^33^,^36^,^50^,^53^,^55^,^60^-^62^	11 (16.42)
Caregiver age^14^,^17^,^18^,^20^,^21^,^43^,^62^-^65^	10 (14.93)
Female^14^,^18^,^20^,^21^,^36^,^39^,^56^,^62^,^66^	9 (13.43)
Lowest educational level^10^,^16^,^21^,^23^,^25^,^35^,^54^,^62^,^64^	9 (13.43)
Restriction social^30^,^31^,^36^,^42^,^52^,^55^,^67^-^69^	9 (13.43)
Lowest family income^10^,^16^,^23^,^26^,^27^,^43^,^53^,^63^	8 (11.94)
Resignation from professional life^17^,^18^,^29^,^30^,^40^,^64^,^69^,^70^	8 (11.94)
Restriction financial^33^,^34^,^40^,^45^,^55^,^69^,^71^	7 (10.45)
Insufficient support^15^,^17^,^34^,^50^,^53^,^55^,^64^	7 (10.45)
Being the main caregiver^17^,^19^,^22^,^23^,^31^	5 (7.46)
Level of complexity of care^26^,^28^,^33^,^48^,^72^	5 (7.46)
Being a spouse^23^,^45^,^64^,^73^	4 (5.97)
Living with the care recipient^21^,^31^,^63^,^73^	4 (5.97)
Number of tasks performed^24^,^26^,^30^,^56^	4 (5.97)
Obligation branch^26^,^43^,^66^	3 (4.48)
Limited access to health services^17^,^21^,^74^	3 (4.48)
Limited engagement with the health system^30^,^39^,^40^	3 (4.48)
Feeling unprepared^28^,^49^,^75^	3 (4.48)
Caregivers singles^50^,^37^	2 (2.99)
Confrontation maladaptive^21^,^39^	2 (2.99)
Family size^35^	1 (1.49)
Lack of preparation for work as caregivers^21^	1 (1.49)
Care demands that exceed resources^52^	1 (1.49)
Lower level of self-efficacy^34^	1 (1.49)
Inadequate knowledge about diseases and treatment^30^	1 (1.49)

*Multiple answers

According to the results, there was recognition of 29 factors that precede the Caregiver's Role Strain, which are related to the caregiver him/herself, the patient or the context of the disease. 

Regarding the individual caregiver, the following stand out: the age of the caregiver (14.93%), being female (13,43%), having a lower educational level (13.43%) and being the primary caregiver (7,46%). Regarding the patient and the illness process, the following stand out: the hours of care spent (29,85%), severity of the illness (28.36%), increase in the patient's functional or cognitive impairment (23,88%) and the type of relationship with the care recipient (17,91%). Therefore, it is possible to recognize the magnitude surrounding the burden of care and its repercussions at the individual and family level. 

**Outline of consequents**

In order to recognize the consequences of the concept, it is necessary to extract from the research results the aspects that may result in the concept. However, it is essential that there is an intentional delineation of positive and negative consequences to the concept, in this case, Caregiver Role Strain^7^. Table 5 highlights the consequences highlighted. 

**Table 5:** Consequences of caregiver role strain according to the Scoping Review. Natal, 2024. (n= 67)

Consequent*	n(%)
Depression^14^,^26^,^28^,^29^,^32^,^33^,^35^,^39^,^40^-^42^,^44^,^48^,^49^,^54^,^60^,^63^,^69^,^73^	19 (28.36)
Anxiety^14^,^26^,^28^,^29^,^32^,^31^,^40^-^42^,^48^,^49^,^54^,^55^,^69^,^70^,^73^,^76^	17 (25.37)
Deterioration of physical health^3^,^10^,^19^,^21^,^26^,^31^,^33^,^37^,^38^,^40^,^41^,^45^,^48^,^64^,^67^,^69^,^70^	16 (23.88)
Sleep deprivation^19^,^25^,^26^,^29^,^32^,^40^,^41^,^53^,^55^,^61^,^69^,^70^,^74^,^77^	14 (20.90)
Caregiver mental health impairment^20^,^25^,^29^,^34^,^48^,^56^,^64^,^69^,^70^,^78^	10 (14.93)
Role conflict^26^,^52^,^53^,^57^,^69^,^78^	6 (8.96)
Fatigue^39^,^53^,^55^,^69^,^70^	5 (7.46)
Sadness^43^,^47^,^55^,^70^	5 (7.46)
Helplessness^39^,^43^,^52^,^57^,^70^	5 (7.46)
Self-blame^40^,^57^,^63^,^78^	4 (5.97)
Perception of ineffective health care^25^,^53^,^74^	3 (4.48)
Poor quality of life^10^,^18^,^41^	3 (4.48)
Neglect of one's own needs^32^,^41^,^55^	3 (4.48)
Loneliness^38^,^69^,^79^	3 (4.48)
Feelings of unhappiness and dissatisfaction^21^,^52^	2 (2.99)
Social isolation^63^,^50^	2 (2.99)
Productivity as an individual and your ability to provide quality care^21^	1 (1.49)
Disorders of the musculoskeletal system^29^	1 (1.49)
Coping strategies^31^	1 (1.49)
Hypervigilance regarding the occurrence of new symptoms^39^	1 (1.49)
Feeling stuck in the role^39^	1 (1.49)

*Multiple answers

There is a predominance of 21 consequences of Caregiver Role Strain, which have distinct characteristics, between positive and negative aspects. However, most of the factors that result in the concept are of a harmful nature, highlighting depression (28.36%), anxiety (25.37%), deterioration of physical health (23.88%) and sleep deprivation (20.90%). As for positive factors, there is a predominance only of productivity as an individual and their ability to provide quality care (1.49%) and coping strategies (1.49%). 

It is worth noting that some consequences or antecedents of the concept Caregiver Role Strain can be seen when the caregiver manifests similar terms mentioned, such as caregiver overload, stress or caregiver burden. Thus, they can sometimes be seen in more than one phenomenon or present themselves as a potential confounder in the recognition of tension. 

**Modeling**

The modeling stage requires the process of defining and identifying examples that can illustrate the concept and its aspects, which may be clinical or research references. A similar model will be presented in order to demonstrate all the relationships evidenced in the analysis of the concept^7^. 

It is worth noting that the model was constructed based on the content found through the review and based on the theoretical and clinical proximity of the research team with the chosen concept. 

*Family caregiver, 68 years old, incomplete high school education, Catholic, widowed, mother of four married children, grandmother, retired for two years. She currently lives with her 96-year-old mother, a recently divorced daughter and her granddaughter, in a rented property located in the interior of Paraíba. She reports that she used to live in another state, but moved four years ago, when her father became ill, who was diagnosed with Alzheimer's and died less than two years ago. Since then, she has taken on the care of her mother, who suffered a stroke a year ago and is confined to bed. She reports having difficulty performing more complex care, such as bed baths and feeding through a nasoenteric tube, since it requires skills to move in bed and to handle food and introduce food. Sometimes, she needs financial support to cover medical or nutritional expenses, since the family income is minimal and is not capable of covering the support of all members of the family context. Because she is part of the Family Health Team (ESF) coverage area, she receives guidance when she has questions about care. However, she remembers that access to health services is difficult, especially when urgent care is needed. Since she started taking care of her mother, her blood pressure has changed, and she has developed multiple comorbidities. She also feels physically tired and complains of body aches due to the frequent effort to change positions in bed. She sleeps little at night, worried about what might happen. She misses leisure, social activities and physical activities, especially walking. She has not attended church for a long time, and this bothers her a lot, as she was very attached to religious activities. But she says that, despite having given up many aspects of her life, she has no doubt that she needs to take care of her mother.*

According to the model case presented, it is possible to visualize the nuances faced by a caregiver in the care process, therefore, it becomes more feasible to recognize the manifestation of Caregiver Role Tension after the caregiver assumes this role, particularly due to some antecedents described, namely: Hours of care spent, type of relationship with the care recipient, dependence, insufficient support and the following consequences such as deterioration of physical health, fatigue, perception of ineffective health care and social isolation. 

**Analogy**

In analogy, there is a deliberate choice to describe the concept under development through another phenomenon that is sufficiently similar to what is being studied, but that has been explored more extensively in a more systematic way, thus being better understood and visualized in comparison to the construct highlighted in the study^7^. To this end, the analogy of a mobile cited by Wright and Leahey in 2019^80^ was used. 

*“Visualize a mobile, with four or five parts, suspended and moving smoothly in the air. All in equilibrium. Some parts move rapidly; others are almost stationary… a breeze that barely touches a segment of the mobile immediately influences the movement of each part, some more than others, and then it moves chaotically for some time. Gradually, all exert their influence on the parts and equilibrium is restored, but not until there is a change in the direction of the whole.”*

When considering this definition and its usefulness in relation to care, it is observed that each care context is a unit, the family nucleus, and, consequently, interaction occurs between all members. Thus, the installation of the Tension phenomenon may be related to the perception of isolation and anguish experienced by caregivers, due to the individual loneliness in sustaining the moment of imbalance caused by illness, therefore, it is not possible to reestablish the stable movement of the pieces. 

** Synthesis**

**Figure 2:**
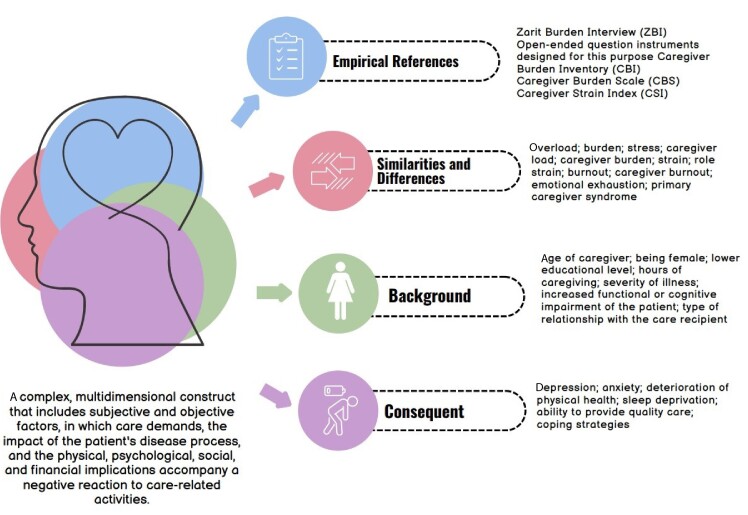
Pictogram of relationships in the Caregiver Role Tension highlighted from the conceptual analysis. Natal, 2024

 The synthesis process involves the gathering of discoveries, meanings and properties that were amplified by each of the conceptual analysis processes^7^. Therefore, a pictogram was constructed to graphically illustrate the main findings of the research, as well as the relationships that surround the concept under study, as shown in Figure 2.

## Discussion

The concepts surrounding caregiver burden have evolved throughout history. It was originally understood as a “cost to the family,” and was understood as something global and one-dimensional. Later, it was understood as two-dimensional, encompassing the objective burdens related to the demands of care and the objective burden related to the caregiver’s attitudes and feelings. Currently, the concept is understood as a multidimensional construct, and therefore includes the physical, psychological, emotional, social, and economic repercussions for the individual who provides care^20^. 

It is noted that caregiver burden is a common concept in health research, and particularly in those related to family and contexts of illness and dependence, caregiver burden and caregiver strain are used interchangeably, as well as other similar terms, due to the similarities presented by their characteristics and contextual applications. Therefore, the lack of clarity in the distinction and definitions for each, which are chosen by the authors, presents itself as a common adversity^10^. 

There are many disciplinary terms like caregiver burden that give rise to confusion. In this sense, caregiver role strain for nursing is understood as a commonly recognized nursing diagnosis, which refers to difficulties in performing the role of informal caregiver. Therefore, its close relationship with the concept of burden is explained, which is widely discussed in the health area in general, as well as in other taxonomies that employ the nursing process. 

It is important to remember that the concepts of tension and stress are also related to the engineering discipline, therefore, with the findings of the conceptual analysis and bringing them to nursing, it is reflected that stress as the events presented externally to care, while tension involves the responses or changes of the individuals who provide care, after being exposed to a network of stressors. Thus, the use of the concept Caregiver Role Strain offers a clearer construct, highlighting the similarities that are present externally to their environment, and that can generate transformations in the health outcomes and quality of life of the caregiver^19^. 

In relation to the empirical references identified, Zarit the Burden Interview (ZBI) is the most widely used scale to measure caregiver strain. This result corroborates a systematic review carried out in 2022, which aimed to investigate the prevalence of burden among caregivers of individuals with mental illness, which recognized the most widely used instruments in inferring the concept under study^21^. 

Other predominant instruments were the Caregiver Burden Inventory (CBI), Caregiver Burden Scale (CBS) and Caregiver Strain Index (CSI). Some of these are validated in other languages, such as the Spanish version of the CBI, which allows understanding the multidimensional perception involving time dependence, developmental, physical, social and emotional overload^77^. 

A study found that the prevalence of caregiver burden measured using the Zarit Burden Interview was higher (36,9%) compared to research using other instruments (26,47%). This can be explained by the fact that the ZBI was proposed with the aim of measuring the burden among different populations and because it has different structures and target populations. Therefore, it is currently considered the main reference for inferring the burden of caregivers and in different contexts of illness^21^. 

Regarding the antecedents found in the conceptual analysis, the hours spent caring predominate. When informal caregivers are involved in caring for a sick person, they assume multiple responsibilities, as well as offering assistance in daily life activities and self-care to patients. Thus, the duration of contact and the provision of long-term care are conducive to the installation of role tension. The entire scenario requires additional hours and commitment to care activities , which contribute to the resignation of the employment relationship and a decline in social or leisure activities for caregivers^30^. 

Other indicators were the severity of the disease and the increase in the patient's functional or cognitive impairment. Particularly in chronic conditions, as the duration of care increases, the severity of these diseases increases concomitantly. As in Parkinson's disease, the patient may be confined to bed, so the caregiver needs to provide additional care, favoring the deterioration of their health in favor of the burden^38^. 

Regarding women, there is a susceptibility to becoming caregivers. The emotional involvement expressed by this population and the marital union, they are expected to fulfill all the duties of mother, wife and daughter, in addition to the obligation to care for their parents. Sometimes, the presence of an employment relationship is even more aggravating, given the multiple facets of the roles assumed by them. And in most cases, those who have no education, are unemployed and in the lower socioeconomic classes, establish a tendency to face a greater economic crisis compared to men, while playing the role of caregiver converging to the incidence of the burden^18^,^39^. 

Caregiver role strain is related to various aspects related to the caregiver, the care recipient, or relational and contextual factors. The characteristics manifested by caregivers lead to harm to physical and mental health, among which high rates of anxiety and depression stand out as consequences of the phenomenon. Therefore, the coping strategies presented by caregivers are associated with and mediate the burden condition^31^. 

A study conducted in the United Kingdom found a high incidence of anxiety and depression reported by informal caregivers. Presumably , this is due to a set of experiences lived by these people, such as the emotional impact of caring for a patient with progressive and limiting diseases, financial difficulties due to expenses, occupational limitations or the impact of the time spent performing the associated care tasks^32^. 

A cohort study conducted with informal caregivers also found incidence rates of 63.5% and 34% of anxiety and depression in patients with Chronic Obstructive Pulmonary Disease (COPD), due to the worsening of the disease. Consequently, the findings suggest that the manifestation of these diseases linked to the worsening of the illness may act in a bidirectional manner, that is, the progression of the disease causes high levels of anxiety and depression in the caregiver, and those individuals who are more depressed and anxious adhere less to the recommended treatments, contributing to the worsening of the illness^81^. 

Other observable consequences were the deterioration of physical health and sleep deprivation. A cross-sectional study conducted in Serbia demonstrated that the reduction in the physical health of caregivers, the neglect of their health, and the abandonment of physical exercise due to the constant demand for care were the result of overload. Therefore, being healthy is a determining variable in the manifestation of stress^40^,^70^. 

Family members who are diagnosed with chronic diseases occasionally take on informal care, and the poor conditions they experience lead to more obvious physical burdens. They often report loss of appetite and apparent fatigue, which may be due to the daily activities related to caregiving. The interruption of their routine leads to physical overload, with sleep disorders, loss of appetite, and lack of energy. Those with chronic conditions in particular are likely to feel overwhelmed by the deterioration of their physical conditions that prevent them from providing care^53^. 

In summary, caregiver role strain can lead to outcomes for informal caregivers and care recipients, such as worsening symptoms, cognitive impairment, decreased quality of life, and physical, psychological, social, and financial aspects of both, making the experience more stressful, decreasing self-efficacy, and lower subjective well-being. In addition, many caregivers who take on the role of care provider may not have had any adequate training or education in providing care and therefore do so informally. In this case, the illness would therefore have an influence not only on the person with the disease, but also on those around them. 

The limitations of this study focus on the types of studies included and available, particularly the absence of longitudinal studies and the reliance on observational studies. Given that the execution of longitudinal and experimental research makes it possible to understand the phenomenon in its entirety, since the chronic or degenerative nature of the context presented by care makes it possible for caregivers to adapt to their role over time or for the disease to exacerbate, short-term studies may make this estimate unfeasible. 

## Conclusions

The findings of the conceptual analysis proposed a theoretical definition for Caregiver Role Strain, operational definitions in order to recognize the phenomenon in care through the recognition of 39 instruments, the differentiation between similar terms such as overload, burden, stress and caregiver load, the delineation of the 29 antecedents (conditions in which the concept is perceived) and 21 consequents (conditions that result in the concept) of the environment and in order to describe the phenomenon in the practical context, the exemplification by the model case and an analogy. 

The proposed method was useful to facilitate a comprehensive understanding of the concept and the relationships in its surroundings and in this population, even though the phenomenon occurs in different contexts, such as chronic, mental and degenerative diseases, or in the care of elderly individuals, dependents and children. Thus, it is important to support the construction and its meaning, design and develop theoretical knowledge as support so that it can be related and meet the needs of caregivers, as well as favor the instrumentalization in research and clinical practice. 

Finally, the study contributes to enabling a comprehensive understanding of caregiver role strain, clarifying its theoretical concept and recognizing its manifestation based on empirical references capable of measuring it. For all these reasons, the analysis implies the development of theoretical and scientific gradients to improve nursing care in recognizing the phenomenon, which presents itself as a complex and multidimensional system, in which each element can predict strain, and is sometimes not recognized in care practice and contributes to the deterioration of the physical and mental health of caregivers. 
